# The Influence of Modification with Natural Fillers on the Mechanical Properties of Epoxy Adhesive Compositions after Storage Time

**DOI:** 10.3390/ma13020291

**Published:** 2020-01-08

**Authors:** Izabela Miturska, Anna Rudawska, Miroslav Müller, Petr Valášek

**Affiliations:** 1 Department of Production Engineering, Faculty of Mechanical Engineering, Lublin University of Technology, Nadbystrzycka 36, 20-618 Lublin, Poland; a.rudawska@pollub.pl; 2 Department of Material Science and Manufacturing Technology, Faculty of Engineering, Czech University of Life Sciences Prague, Kamýcká 129, 165 21 Prague, Czech Republic; muller@tf.czu.cz (M.M.); valasekp@tf.czu.cz (P.V.)

**Keywords:** fillers, epoxy resins, modification, SEM

## Abstract

This article presents the initial test results examining basic technological factors, such as type of modifying agent and seasoning time, which influence properties of adhesive epoxide compositions. The aim of the study was to prepare adhesive compositions with 2% content of the selected natural fillers (montmorillonite NanoBent ZR-2, ground chalk (powder)—CaCO_3_, and activated carbon powder C) and to examine their strength properties. A polymeric matrix used to prepare an adhesive composition consisted of the epoxide resins used in industry: Epidian 5 and Epidian 53 cured by addition of an aminomethyl group, where curing occurred through the Mannich reaction. A composition of epoxide resins with a curing agent and without any modifying agents was used as reference. The examinations described in the present article aimed to show the significance of the impact of the fillers used on the strength properties of the examined compositions. A fracture surface of epoxide adhesive compositions modified with the selected fillers was tested by means of a scanning electron microscope.

## 1. Introduction

The use of adhesive bonds as an alternative to traditional joining methods is becoming more and more popular. It results, among others, from the fact that polymer composite materials are being used in new construction projects to a larger scale now [1,2]. As a result, more and more research is being conducted in order to improve their strength properties. However, such research is conducted not only in order to improve the strength properties themselves, but also to gain more performance characteristics [3,4,5], such us increased fatigue life and static durability, better thermal connectivity, fire retardancy or improved electric and thermal conduction, as well as water absorption. This research is conducted in several directions [6,7,8,9,10]: technological, constructional, and material. In addition, a direction related to modifications of adhesives and adhesive-bonded joints is taken into consideration more and more often, and it is aimed at improving the properties of the adhesive bonds themselves. It should be emphasized that the strength properties of the adhesive bonds depend on numerous factors. The most important ones are the type of adhesive composition used, the preparation technique of the surface of the bonded parts, the conditions of the adhesive-bonded joint curing, and the construction of the adhesive bond itself [11,12,13]. By modifying the adhesive compositions, it is possible not only to improve adhesive bond strength, but also to gain or improve other properties, e.g., resistance to some hazardous use factors. One of the modification types is physical modification that involves adding some appropriate compounds, such as fillers, modifiers, or modifying agents [14,15].

Epoxide resins are one of the most popular types of matrices used for the preparation of adhesive compositions that are, in turn, used as structural adhesives in many different industrial sectors, e.g., aviation, automotive, construction, maritime, and machine construction industries, or as protective coatings [16,17]. This is due to numerous factors, including very good adhesion to a wide range of materials, relatively high strength in the cured state (both static and fatigue), low curing shrinkage, and resistance to numerous exploitation factors [13,18,19]. In order to modify the properties of the epoxide compositions, different additives are added [20,21,22,23,24]. The exemplary modifying substances are diluents, supplying agents, pigments, colors, softening agents, antioxidants, stabilizers, and fillers [20,22,25]. The epoxide resin fillers that are widely used are powdered and granulated copper, aluminum, corundum, aluminum silicate, chalk, silica, glass, graphite, quartz, metal fibers, carbon nanotubes, and carbon black [16,26,27]. Adhesive compositions that are modified by the addition of fillers are stronger and adhesive bonds are more durable by 25–30% [28,29]. According to the references [2,8,9,10,26,27,28,29,30] it can be stated that, in terms of adhesive compositions, the degree of mechanical or physical property changes caused by the addition of fillers depends on numerous factors, such as a filler type, quantity, mixing conditions, or type of the basic resin and curing agent [20,31,32,33,34]. Adding fillers to the epoxide resins brings positive effects on some properties of the cured material. In some cases, it also facilitates the material’s processing, which in turn prolongs the composition’s life and decreases the exothermic effect of the crosslinking reaction and, in many cases, decreases costs. Modifying agents used in the study were three types of fillers, namely montmorillonite NanoBent ZR-2, ground chalk (powder), and activated carbon powder.

One of the important fillers is organic montmorillonite (M*_x_*(Al_4−*x*_Mg*_j_*)(Si_8_)O_20_(OH)_4_) [4]. The montmorillonite plate is 0.96 nm thick. The length of its other dimensions is about 200–1000 nm. Particular plates are bound by Van der Waals forces, taking into consideration that the distance between two consecutive plates is about 0.3 nm. The sum of plate thickness and the distance between the two consecutive plates is called the basic dimension and amounts to 1.26 nm. Five to ten parallel plates bound by van der Waals forces constitute a montmorillonite initiating particle of a total thickness of 7–12 nm. These particles form agglomerates sized 200–1000 nm [7,8,34]. 

An inorganic filler that is gaining more and more popularity is chalk. It is a sedimentary rock, which is a form of limestone (CaCO_3_) that does not contain numerous admixtures. Due to its properties, chalk is widely used in ceramic, chemical, pharmaceutical, and cosmetics industries. It is also often used in the production of white paints, powders, and toothpastes. The usage of chalk as a modifying agent in appropriate proportions that, according to the references, ranges between 2 and 8 weigh particles, improves impact strength and resistance to bending and compression [35,36]. The addition of chalk improves physical characteristics, especially thermal stability [37,38].

Another organic filler that was used in the study was activated carbon. Production of the activated carbons is based on natural organic raw materials of polymeric composition [16]. In the studies examining the modified epoxide resins, activated carbon fillers used so far were in the form of graphite nanoparticles, carbon nanotubes, graphite powder, coke coal, hard coal, glassy carbon, as well as carbon fibers [39,40]. Carbon fillers in the amorphous form—carbon black—have also been used recently [26]. Addition of carbon fillers in epoxide resin compositions improves impact, peel, and compressive strength. Moreover, physical characteristics are improved as well, including higher thermal diffusivity, lower liquid permeability, lower linear thermal expansion coefficient, and higher thermal conductivity [39,40].

The study results shown in the present article include parts of a study concerning modification of the epoxide adhesive compositions. Due to a variety of the modifying agents and adhesive compositions, as well as different impact of the modifying agents on the adhesive compositions’ characteristics, the aim is to conduct research on this issue, not only for cognitive reasons, but also for potential application purposes.

It seems to be interesting to check whether the type of the filler used, and the storage time have a vital influence on the strength properties of the adhesive compositions. The aim of the article was to study the mechanical properties of epoxide adhesive compositions modified with the use of natural fillers after seasoning in selected conditions. A fracture surface of epoxide adhesive compositions with the fillers montmorillonite NanoBent ZR-2, ground chalk (powder)—CaCO_3_, and activated carbon powder C was also tested using a scanning electron microscope.

## 2. Materials and Methods

The aim of the study was to prepare and determine strength properties of epoxide adhesive compositions with 2% content of the selected natural fillers. The polymeric matrices used to prepare adhesive compositions consisted of the epoxide resins used in industry, namely Epidian 5 and Epidian 53 resins, cured by addition of an aminomethyl group (a TFF curing agent), where curing was carried out by the Mannich reaction.

### 2.1. Epoxide Resins

**Figure 1:**
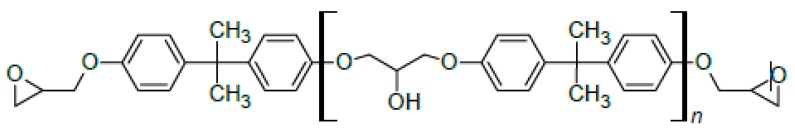
Structure of Epidian 5 epoxy resin [41].

Epidian 5 (producer: CIECH Sarzyna, Nowa Sarzyna, Poland) is a pure form of epoxide resin, which is a product of the reaction of bisphenol A with epichlorohydrin. It is characterized by high adhesiveness (at 25 °C: 15,000–30,000 mPa·s) and density (at 20 °C: 1.18–1.19 g/cm^3^). Additionally, it shows great adhesion to the majority of materials, chemical resistance, resistance to aggressive environmental factors, and good electric properties [20,22,40]. Epidian 5 and its modifications are used in the production of laminates made of glass fiber, as well as in welding metals, ceramics, and thermocuring materials. Epidian 5 based adhesives are also used in construction works as anticorrosion and electro-insulating coatings. Structure of Epidian 5 epoxy resin is shown in Figure 1.

Epidian 53 (producer: CIECH Sarzyna) is a mixture of the epoxide resin made of bisphenol A and epichlorohydrin (Epidian 5) and styrene. It is characterized by low adhesiveness (at 25 °C: 900–1500 mPa·s) and lower density than Epidian 5 (at 20 °C: 1.11–1.15 g/cm^3^). Epidian 53 is characterized by high strength at a temperature of about 110 °C [20,22,42]. Its modifications are used in joining glass laminates. Due to great electro-insulation and resistance properties, it can be used in radio engineering, aviation, and optics.

Characteristics of the epoxide resins used in the study are presented in Table 1.

### 2.2. Curing Agent

Curing of the epoxide resins was carried out after adding the TFF (the Mannich reaction) curing agent in appropriate proportions. Proportions of the epoxide resins and curing agent in the epoxide resins compositions that are recommended in the stoichiometric quantity [44] and that were used in the studies are presented in Table 2. The TFF curing agent is used for curing the epoxide compositions for construction works, where working conditions often include low temperatures and high humidity. Due to good chemical resistance of such compositions in different types of aggressive environments, the TFF curing agent is qualified for curing anti-corrosion linings in industry. A positive hygienic assessment makes it possible to apply the TFF curing agent in different types of epoxide coatings in the public utility areas and in the food industry. The TFF curing agent is characterized by high adhesiveness (at 25 °C: 10,000 mPa·s) and medium density (at 20 °C: 1.15–1.20 g/cm^3^) [20].

Due to high reactivity of the TFF curing agent, the mixture of the resin and the curing agent was prepared directly before use in the proportions possible to be used within a few minutes.

### 2.3. Fillers

Modifying agents used in the study were of the following three types of fillers: montmorillonite NanoBent ZR-2, ground chalk (powder), and activated carbon powder. Information about the fillers used is presented in Table 3. The fillers were selected due to their origin and environmental aspects. They are natural materials, derived from natural deposits. The environmental protection during the process of preparing construction materials for technology is of high importance and relevance. This is why the use of natural fillers seems to be an interesting concept. 

A filler of highly fragmented structure forms the nanosilicates group, under the NanoBent ZR-2 trade name, produced by ZGM “ZĘBIEC” SA (a mining–metal plant in Poland). ZR-2 NanoBent is an aluminosilicate modified by the quaternary ammonium salt. It can be used as a double-acting (thixotropic and biocidal) additive. As a thixotropic additive, it improves the functional characteristics of the composition [45]. 

Another filler used in the study was ground chalk produced by “KredKop” (Rabka-Zdrój, Poland). Chalk is a soft sedimentary rock of relatively soft and porous texture, which influences its specific gravity.

The last filler was activated carbon in a powder and fine-crystalline form. It has strong adsorption capacity, as well as strongly developed specific surface area, which stems from the inner porous structure. It is a cheap and non-toxic substance.

### 2.4. Preparation of Epoxide Compositions’

**Figure 2:**
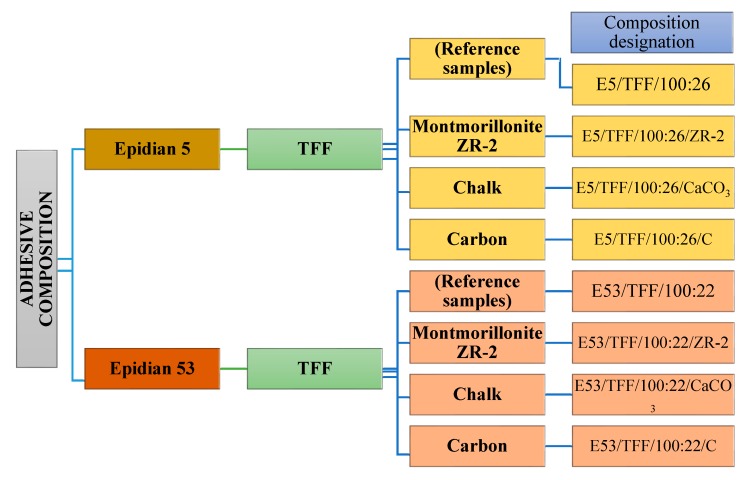
Scheme of the adhesive compositions’ ingredients used in the study and their designations.

The subjects of the study were 8 variants of the epoxide adhesive compositions in a cured state based on Epidian 5 and Epidian 53 epoxide resins, cured with the TFF (Table 2) and modified with the following fillers: Montmorillonite ZR-2, chalk CaCO_3_, and carbon powder C in a 2% mass ratio (filler to resin). The scheme of the epoxide adhesive resins used in the study is presented in Figure 2.

For the purposes of comparison, compositions of the epoxide resins with a curing agent and without any modifying agents were used. The adhesive compositions were prepared with the use of a chemical mixer with a special propeller bi-panel mixer arm that made it possible to disperse the filler in the whole volume of the epoxide resin. The mixing speed was 460 rpm, and the mixing time was 3 min. Then the curing agent was added to the modified resin in the appropriate weight ratio. The adhesive composition was then subjected to the process of gas build-up removal.

### 2.5. Shape and Dimensions of Samples

**Figure 3:**
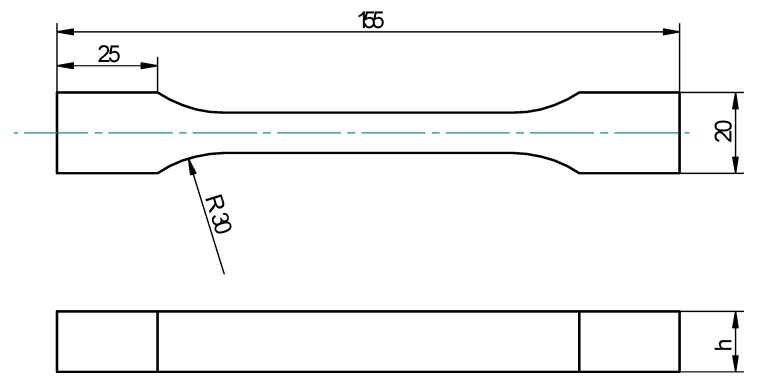
Geometry and dimensions of the prepared epoxide compositions sample.

The samples, whose shape and dimensions are presented in Figure 3, were cast in a special silicone form, due to which it was possible to achieve the sample shape specified in the PN-EN ISO 3167:2014-09 standard [46]. The silicone form was coated with an antiadhesive agent before preparing the epoxide compositions samples. Due to this it was possible to take the cured adhesive compositions out of the form.

Figure 3 shows the shape and dimensions of the prepared casts. The ‘h’ was a variable dimension within the specified limits and measured for every sample separately directly before strength tests. It was 4 ± 0.2 mm. Strength tests of the cured samples were performed on the Zwick/Roell Z150 testing machine, (Zwick/Roell, Wroclaw, Poland), in accordance with the norm related to determination of the static tensile mechanical properties of the cured materials—PN-EN ISO 527-1:2012 [47].

### 2.6. Curing and Storage Conditions

Preparation, curing, and storage conditions of the epoxide compositions were performed and cured in the laboratory conditions at temperature of 25 °C ± 1 °C and 27% ± 1% relative humidity. Ten samples of every composition were prepared. The cured samples were stored in cardboard boxes to avoid exposure to sunlight. Table 4 presents the designation of the samples.

The samples were left for curing for 7 days. Then, according to the study plan, a part of the cured samples of the adhesive compositions were subjected to the strength test, which was aimed at indicating tensile strength. The other samples were seasoned for four months in the same storage conditions as those in which the adhesive compositions had been prepared. Then a strength test was performed. Due to this it was possible to perform a comparative analysis of the results and to determine the impact of the storage conditions on the strength properties of the compositions. 

The study included the use of 10 samples in each group in order to indicate the standard deviation of strength and to conduct a statistic comparative analysis of the results, given the materiality level α = 0.05.

## 3. Results

The results of the strength tests for each group of samples, together with statistical analysis, is presented below.

### 3.1. Test Results of the Adhesive Compositions from the E5/TFF/1 Week Group

Table 5 shows results of the strength tests of the adhesive compositions from the E5/TFF/1 week group (according to Table 4).

Based on the results presented in Table 3, it can be stated the adhesive composition E5/TFF/100:26 with the addition of chalk showed the highest strength. At the same time, the elongation value was also the highest for this composition (2.72%). The lowest strength and elongation value were obtained for the composition with the addition of NanoBent ZR-2. For the adhesive composition with the addition of carbon, the strength was lower than for the reference sample (unmodified composition) but the elongation value in tension was higher.

However, in order to assess the obtained results completely, it was necessary to conduct a statistical analysis. The assumption of normal distribution (Shapiro–Wilk test) and of variance homogeneity (Levene test) proved correct. In this context, the impact of fillers on the strength properties of the adhesive compositions was tested.

All results were compared with each other with the use of the Tukey’s test (ANOVA). The results are presented in Table 6.

Based on the obtained results, it could be observed that at the assumed significance level α = 0.05 the results did not vary significantly (*p* value > α). 

Based on the results presented in Table 5 and Table 6, it could be observed that there was no statistically significant difference between the strength value between the E5/TFF/100:26 modified adhesive compositions. This was proved by the fact that the results of each group were in one homogenous group designated by the post-hoc test.

### 3.2. Test Results of the Adhesive Compositions from the E53/TFF/1 Week Group

Table 7 shows the results of the strength tests of the adhesive compositions from the E53/TFF/1 week group (according to Table 4).

Strength test results of the adhesive compositions from the E53/TFF/1 week group presented in Table 7 indicate that the highest strength (16.84 MPa) and elongation value (3.08%) were obtained in the case of the reference samples without any modifier. Compositions modified with carbon obtained a bit lower strength (15.32 MPa) and elongation in tension (2.29%). Similar elongation was obtained for the compositions modified with chalk (2.31%) in spite of lower strength (13.55 MPa). Moreover, in case of the compositions modified with chalk, the reproducibility of testing results was the best. In order to assess significant differences, statistical analysis of the results was conducted. The assumption of normal distribution was fulfilled but the Levene’s test allowed us to reject the assumption of variance equality. Due to this fact, a rank non-parametric Kruskal–Wallis test was conducted. Those results are presented in Table 8.

When performing the Kruskal–Wallis test, the Pearson coefficient (*R*) indicated the level of linear correlation between variables group. Based on the statistical test (conducted at the assumed significance level α = 0.05) it was observed that there were significant differences between the obtained results. 

Based on the obtained results it could be stated that there were some differences between the examined groups of adhesive compositions, although these differences were not big. Based on the test of significant differences, two homogenous groups were isolated. One of the examined samples (E53/TFF/100:22/CaCO_3_/1W) was assigned to two homogeneous groups. Significant differences were then observed between reference samples of the adhesive compositions modified with carbon and samples of compositions modified with montmorillonite. 

### 3.3. Test Results of the Adhesive Compositions from the E5/TFF/4 Month Group

Table 9 shows the results of the strength tests of the adhesive compositions from the group E5/TFF/4 month (according to Table 4).

Based on the strength test results of the adhesive compositions E5/TFF/100:26 that were seasoned for 4 months, it was observed that the highest strength was obtained for the reference samples (34.35 MPa), although they showed the highest dispersion of results.

In order to check the significant differences between the results, a statistical analysis was conducted, as in the previous cases. The assumption of normal distribution and variance equality did not prove correct, so the Kruskal–Wallis test was conducted. Those results are presented in Table 10.

In the comparison test at the significance level α = 0.05, after setting up a correlation coefficient *R*, it was observed that there was a weak correlation between the strength of adhesive samples modified with chalk and carbon and the reference samples. This means that the results were significantly different. 

Based on the test results and the homogenous groups’ designation, there were significant differences between consecutive groups of samples. After summarizing the strength test results of the adhesive compositions from the E5/TFF/4 month group, it could be stated that there were significant differences between the group of the modified and the reference samples. Reference samples showed significantly higher tensile strength after seasoning time (4 months) in relation to the modified samples. In comparison with the E5/TFF/100:26/C/4M samples, the difference was 24.15 MPa, which was 70% of the strength of the E5/TFF/100:26/4M samples.

### 3.4. Test Results of the Adhesive Compositions from the E53/TFF/4 Months Group

Table 11 shows the results of the strength tests of the adhesive compositions from the E53/TFF/4 month group (according to Table 4).

Based on the results presented in Table 11, it was observed that the reference samples showed the lowest strength (25.19 MPa). However, this result was not unequivocal as the standard deviation from the mean value was very high. In order to assess the differences between the results, it was necessary to make statistical calculations. The distribution of results was in accordance with the normal distribution. However, the assumption of the variance homogeneity was not fulfilled. Due to this fact, a non-parametric Kruskal–Wallis test was conducted. Its results are presented in Table 12.

Based on the test results, it could be observed that there were significant differences between the samples of the adhesive compositions modified with carbon and reference samples of those modified with NanoBent ZR-2. 

Based on results presented in Table 11 and Table 12, where the homogeneous groups were designated, a conclusion drawn based on the rank test’s results was confirmed. This means that there were significant differences at the assumed significance level α = 0.05 between the samples of the E53/TFF/100:22/C/4M and E53/TFF/100:22/4M compositions. It was also observed that the strength results of the compositions modified with chalk were included in the same homogenous group as the adhesive compositions modified with carbon. This means that the obtained strength results did not differ significantly.

### 3.5. SEM Analysis

A fracture surface of epoxide adhesive compositions in the cured modified state, seasoned for four months with the fillers montmorillonite NanoBent ZR-2, ground chalk (powder)—CaCO_3_, and activated carbon powder C, was tested by means of a scanning electron microscope TESCAN MIRA 3 GMX SE detector (SEM) (Tescan Orsay Holding, Brno – Kohoutovice, Czech Republic). The accelerating voltage was 10 kV. The samples were dusted with gold by means of the equipment Quorum Q150R ES (Qourum Technologies Ltd., Laughton East Sussex, England)—sputtering deposition rate using gold.

**Figure 4:**
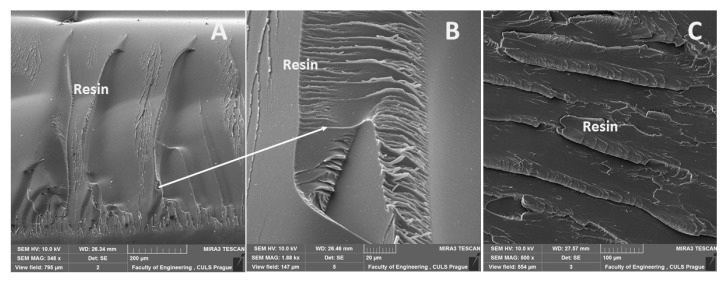
SEM micrographs of adhesive without filler (matrix): (**A**) brittle fracture of adhesive E5/TFF/100:26 (mag 348×), (**B**) detailed view of fracture surface of adhesive E5/TFF/100:26 (mag 1.88k×), (**C**) brittle fracture of adhesive E53/TFF/100:22 (mag 500×).

An interaction and a shape of the filler were obvious from the results of SEM analysis of the fracture surface of the epoxide adhesive compositions (matrices) and the composite materials. In the case of the fracture surface of the adhesive without the filler (matrix), it was a brittle fracture (Figure 4). A detail of the brittle fracture with a specific fracture surface of the adhesive E5/TFF/100:26 is visible from Figure 4B.

**Figure 5:**
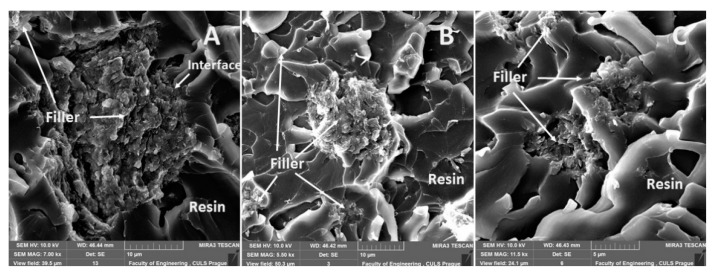
SEM micrographs—static tensile test of composite materials: (**A**) fracture surface of adhesive E5/TFF/100:26/ZR-2 (mag 7.0k×), (**B**) fracture surface of adhesive E5/TFF/100:26/ZR-2 (mag 5.5k×), (**C**) detailed view of fracture surface of adhesive E5/TFF/100:26/ZR-2 (mag 11.5k×).

**Figure 6:**
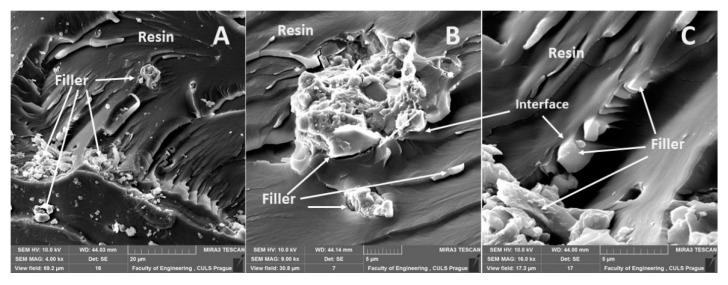
SEM micrographs—static tensile test of composite material: (**A**) fracture surface of adhesive E5/TFF/100:26/CaCO_3_ (mag 4.0k×), (**B**) detailed view of fracture surface of adhesive E5/TFF/100:26/CaCO_3_ (mag 9.0k×), (**C**) detailed view of fracture surface of adhesive E5/TFF/100:26/CaCO_3_ (mag 16.0k×).

**Figure 7:**
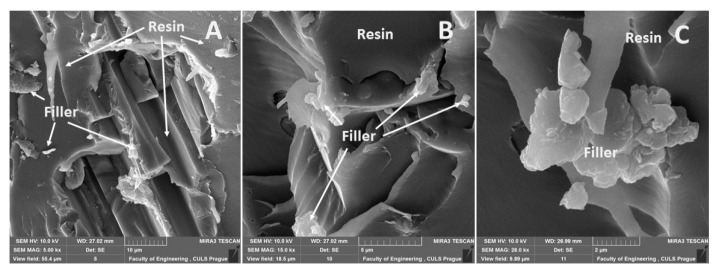
SEM micrographs—static tensile test of composite materials: (**A**) fracture surface of adhesive E5/TFF/100:26/C (mag 5.0k×), (**B**) fracture surface of adhesive E5/TFF/100:26/C (mag 15.0k×), (**C**) fracture surface E5/TFF/100:26/C with detailed view of filler carbon powder (mag 28.0k×).

**Figure 8:**
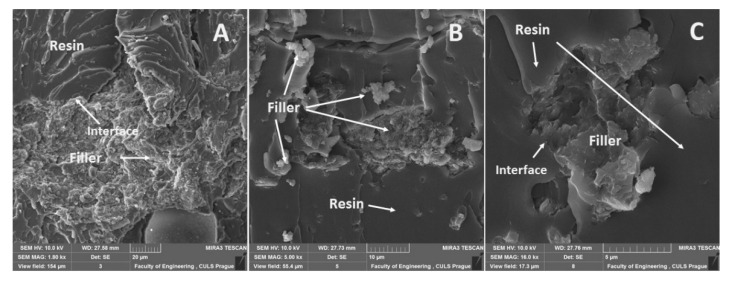
SEM micrographs—static tensile test of composite materials: (**A**) fracture surface of adhesive E53/TFF/100:22/ZR-2 (mag 1.8k×), (**B**) fracture surface of adhesive E53/TFF/100:22/ZR-2 (mag 5.0 k×), (**C**) fracture surface of adhesive E53/TFF/100:22/ZR-2 with detailed view of filler ZR2 (mag 16.0 k×).

**Figure 9:**
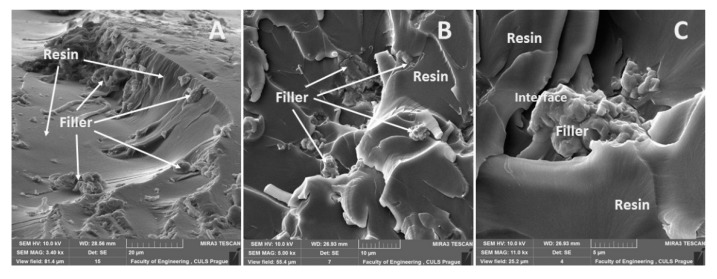
SEM micrographs—static tensile test of composite materials: (**A**) fracture surface of adhesive E53/TFF/100:22/CaCO_3_ (mag 3.4k×), (**B**) fracture surface of adhesive E53/TFF/100:22/CaCO_3_ (mag 5.0k×), (**C**) fracture surface of adhesive E53/TFF/100:22/CaCO_3_ with detailed view of filler CaCO_3_ (mag 11.0k×).

**Figure 10:**
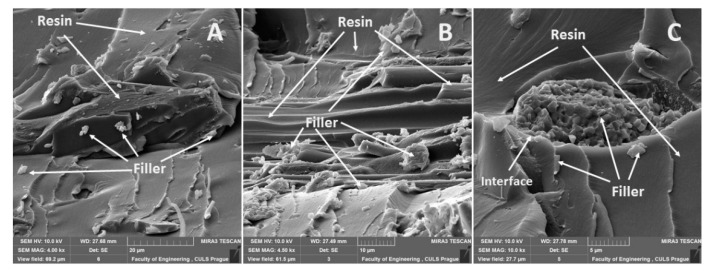
SEM micrographs—static tensile test of composite materials: (**A**) fracture surface of adhesive E53/TFF/100:22/C (mag 4.0k×), (**B**) fracture surface of adhesive E53/TFF/100:22/C (mag 4.5k×), (**C**) fracture surface of adhesive E53/TFF/100:22/C with detailed view of filler carbon powder (mag 10.0k×).

A distribution of particular fillers, their size, shape, and the sizes in interaction with the matrix, i.e., the adhesive E5/TFF/100:26 and E53/TFF/100:22, is visible from SEM micrographs in Figure 4, Figure 5, Figure 6, Figure 7, Figure 8, Figure 9 and Figure 10. 

The fracture surface of the composite material E5/TFF/100:26/ZR-2, i.e., with the filler based on the montmorillonite plate, is visible in Figure 5. SEM analysis showed huge filler size variations, which are evident from Figure 5A–C. The interaction of the filler and the matrix was good. It is obvious from the results of the SEM analysis that the filler was composed of smaller segregated portions distinguished by an irregular shape (plate shape).

The fracture surface of the composite material E5/TFF/100:26/CaCO_3_, i.e., with the filler based on CaCO_3_ microparticles (chalk), is evident from Figure 6. An uneven distribution of the filler in the matrix, which is usual for particle composite systems, is obvious from Figure 6A. A detailed view of the interaction between the filler and the matrix is evident from Figure 6B. A good wettability of the filler with the matrix is visible in Figure 6B,C. A good interaction of the filler and the matrix is a basic presumption of the composite material success [48,49]. 

An overview of the micrographs of the fracture surface is seen in Figure 7A,B, which represents the fracture surface of the composite E5/TFF/100:26/C and the interaction of the matrix and the reinforcing phase. A detailed view of the filler carbon powder is evident from Figure 7C. It is obvious from Figure 7C that it led to delamination of the filler from the matrix.

The fracture surface of the composite material E53/TFF/100:22/ZR-2, i.e., with the filler based on montmorillonite, is evident in Figure 8. A detailed view of the interaction between the filler and the matrix is visible in Figure 8A–C from which good wettability in the interface of the filler and the matrix is obvious. SEM analysis proved increased variability of the filler size, i.e., the difference between Figure 7C and Figure 8A. 

The fracture surface of the composite material E53/TFF/100:22/CaCO_3_, i.e., with the filler based on CaCO_3_ powder (chalk), is evident from Figure 9. The fracture surface of the composite material E53/TFF/100:22/CaCO_3_, i.e., the distribution of the filler in the composite, is visible from the overview in Figure 9A. A more detailed view of the interaction between the filler and the matrix is presented in Figure 9B,C from which good wettability in the interface of the filler and the matrix is obvious. 

The fracture surface of the composite material E53/TFF/100:22/C, i.e., with the filler based on carbon powder, is visible in Figure 10. The overview of the fracture surface of the composite material E53/TFF/100:22/C is evident from Figure 10A,B. It is obvious from Figure 10A,B that the delamination of part of the carbon powder filler occurred at the destruction of the static tensile test. The detailed view of the interaction between the filler and the matrix (resin) is obvious from Figure 10C from which good wettability of the filler with the resin is evident.

## 4. Discussion

**Figure 11:**
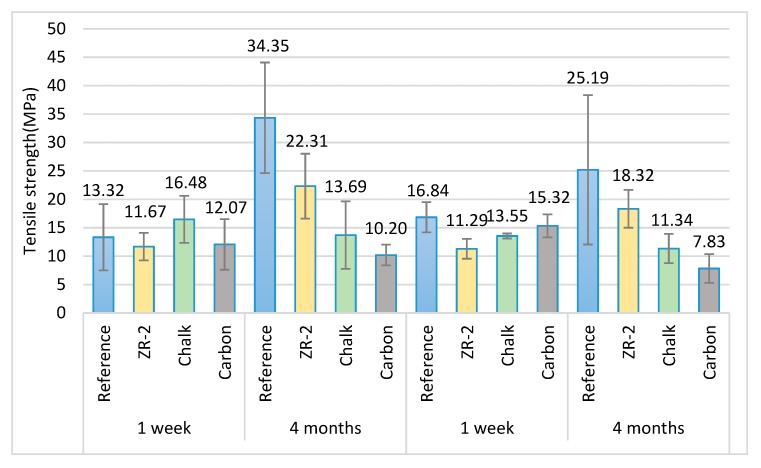
Strength test results of the adhesive compositions subjected to tests.

**Figure 12:**
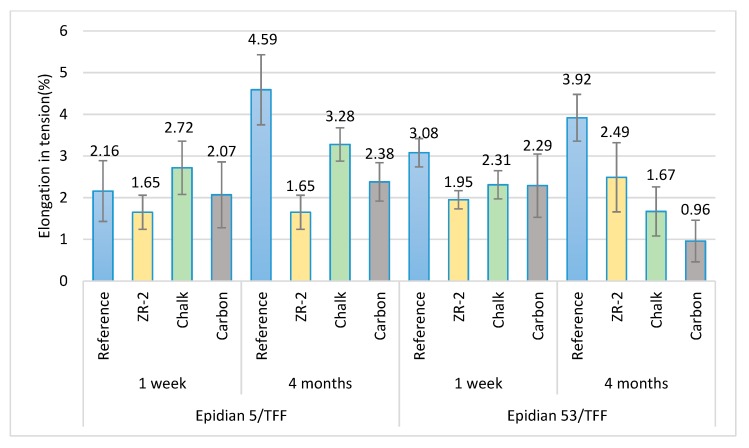
Results of the elongation in tension of the cured adhesive compositions subjected to tests.

In order to conduct a comprehensive comparative analysis of strength of the modified adhesive compositions after a short-term seasoning, the results are presented in Figure 11 and Figure 12.

Based on the results described above, it can be observed that for both compositions subjected to tests, the highest strength was obtained for the reference samples that were seasoned for 4 months. The lowest strength, in turn, was obtained for the adhesive E53/TFF/100:22 composition modified with carbon that was seasoned for 4 months. It can also be observed that in the case of the reference samples for the compositions modified with NanoBent ZR-2, their strength increased over the seasoning time. In the case of the compositions modified with chalk and carbon, the strength decreased.

A corresponding distribution of results was also observed in the analysis of elongation (Figure 12) that occurred during the tensile strength tests. 

**Figure 13:**
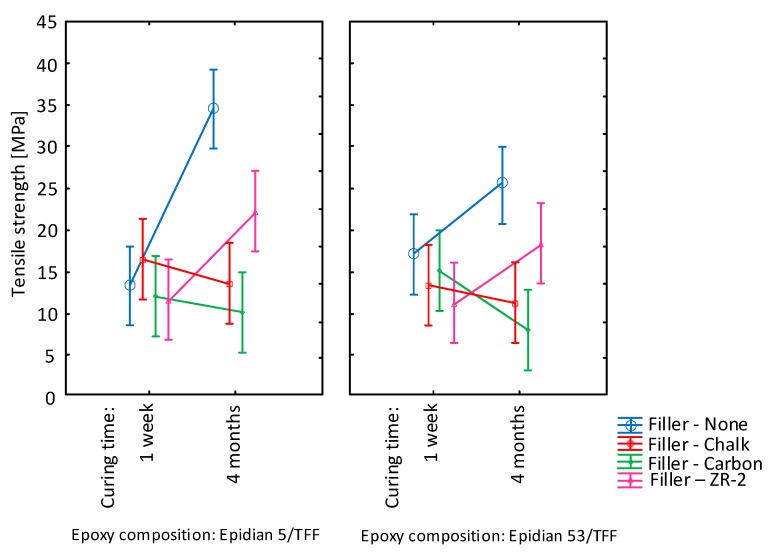
Graphs of interactions on the tested epoxide compositions including the qualitative factors: curing time and filler (vertical bars represent confidence intervals 0.95).

Similar courses of strength considering the seasoning time were obtained for both variants of the basic compositions subjected to tests. They are presented in Figure 13.

Based on the graphs of interaction of the seasoning time and the type of filler, it can be observed that addition of chalk and carbon did not improve the tensile strength of the compositions E5/TFF/100:26 and E53/TFF/100:22. In the case of the latter, the addition of carbon worsened the strength results over time. Taking into consideration the one-week curing of the adhesive compositions it can be observed that the compositions of the Epidian 5 epoxide resin showed better strength characteristics when modified with NanoBent ZR-2 and chalk. For both compositions the best result was obtained with addition of NanoBent ZR-2, whereas the worst was with addition of carbon. 

Significant changes were visible only at the matrix (marked as Reference). The change occurred at the modified adhesive. However, this was insignificant in most cases because of the overlapping of results by error bars (i.e., the standard deviation). However, the change after 4 months was caused mainly by secondary hardening. This trend was obvious, especially at the matrix (Reference). General pieces of information from producers define the hardening time as the time to reach the manipulating strength and subsequent functional strength, but this time is considerably different from reaching full secondary strength, which literary resources state up to several months according to the adhesive type. For example, the publications of Messler and Müller [50,51] deal with this topic. Balkova et al. investigated strength characteristics of the adhesive during hardening for a 1 year time period with the aim of evaluating possible aging influences. They found in their study that the strength of adhesive bonds kept under the laboratory temperature mildly increased over time [52].

It is important to take into account with respect to already created bonds the fact that mechanical properties of adhesive bonds change over time [53,54]. At present, there is no available technique for adhesive bond creation that can predict the environmental degradation of adhesive bonds [55]. Most experience with determining the bond failure mode comes from research on the mechanical properties and fracture surfaces of the bond after its failure [56]. 

The tensile-elongation increase is in accordance with analogous results, e.g., with polypropylene/coir fiber composites treated in NaOH where the increase of the tensile-elongation was also observed [57]. Results of SEM analysis proved good wettability between the filler and the matrix (the Reference), which gives a presumption of full utilization of mechanical properties of the filler, i.e., also increasing the tensile-elongation. 

The increase of the tensile strength and the tensile-elongation is usually problematically reached at the natural filler in the area of the polymeric composites where bad wettability with the matrix is frequent and the surface has to be activated chemically or by plasma [48,58,59].

The results presented in Figure 13 indicate lower strength for the composition of Epidian 53 with the TFF curing agent that was seasoned for 4 months. It is probably connected with the presence of styrene monomer in the resin itself. 

## 5. Conclusions

This paper deals primarily with the influence of the storage time. This experimental procedure depends on requirements of the practical application and connecting storage process. Both studies and experiments allow us to draw the following, most vital conclusions:

- The obtained repeatability of the results of strength of the adhesive compositions indicates the need for improvements in the way adhesive compositions are prepared. Maybe the change of the mixing and degassing methods would allow one to obtain better repeatability of results that may, in turn, prove the uniformity of distribution of the filler agglomerates with the polymeric warp.
- The type of filler affects the strength of the tested compositions over the storage time. In the case of montmorillonite, the strength increases but as far as addition of carbon and chalk is concerned, the strength decreases over the storage time for both cured resin compositions.
- Based on the research, it can be seen that the type of resin used in the adhesive composition also affects the elongation of the composition. It was noted that after 4 months, samples of epoxy compositions with Epidian 53 (chalk and carbon) were getting worst compared with the results for Epidian 5 compositions. Carbon and chalk used as modifiers in the compositions of Epidian5 may be more effective as far as the plasticity coefficient in concerned, as the elongation values were higher. The change of the curing and storage conditions may impact the obtained results.

It must be noticed, though, that this rationale should be confirmed with the modified resins physical properties tests with the use of properly prepared adhesive compositions, as well as with control microscopic examination that would make it possible to assess the distribution of the fillers used in the adhesive compositions’ structure.

The examinations described in the article aimed at showing the significance of the impact of the fillers used on the strength properties of the examined compositions. They also inspired the authors to perform further examinations of a wider scope. We also plan to study the influence of the adhesive compositions’ modification on the strength properties of the adhesive bonds.

## Figures and Tables

**Table 1:** Functional characteristics of the epoxide resins used in the studies (own work based on [20,21,42,43]).

Characteristics	Epidian 5	Epidian 53
Epoxide number (mol/100 g)	0.48–0.52	≥0.41
Adhesiveness in 25 °C (mPa·s)	15,000–30,000	900–1500

**Table 2:** Amount of the TFF curing agent recommended by the supplier and used in the study per 100 g of the epoxide resin [44].

Epoxide Resin (100 g)	TFF Curing Agent Amount (g)
Epidian 5	26
Epidian 53	22

**Table 3:** Characteristics of fillers used in the study.

Characteristics	Montmorillonite NanoBent ZR-2	Chalk	Activated Carbon
Type	Organic aluminosilicate	Inorganic chemical compound in the form of a sedimentary rock	Organic alkaline dust coal, made of charcoal activated with steam
Origin	Natural	Natural	Natural
Form	Light grey solid in the form of powder	White solid in the form of powder	Black powder in the fine crystalline form
Base	Modified by the quaternary ammonium salt	Limestone variety	Activated carbon
Density	<0.5 g/cm^3^	2.711 g/cm^3^	2 g/cm^3^
Molecular formula	M*_x_*(Al_4−*x*_Mg*_j_*)(Si_8_)O_20_(OH)_4_	CaCO_3_	C

**Table 4:** Types of adhesive compositions, seasoning time, and composition designation.

Epoxide Resin	Curing Agent	Filler	Curing Time	Seasoning Time	Designation of a Sample Group	Composition Designation
Epidian 5	TFF	Reference samples	7 days	-	E5/TFF/1 week	E5/TFF/100:26/1W
		Montmorillonite ZR-2				E5/TFF/100:26/ZR-2/1W
		Chalk CaCO_3_				E5/TFF/100:26/CaCO_3_/1W
		Carbon C				E5/TFF/100:26/C/1W
Epidian 53	TFF	Reference samples	7 days	-	E53/TFF/1 week	E53/TFF/100:22/1W
		Montmorillonite ZR-2				E53/TFF/100:22/ZR-2/1W
		Chalk CaCO_3_				E53/TFF/100:22/CaCO_3_/1W
		Carbon C				E53/TFF/100:22/C/1W
Epidian 5	TFF	Reference samples	7 days	4 months	E5/TFF/4 months	E5/TFF/100:26/4M
		Montmorillonite ZR-2				E5/TFF/100:26/ZR-2/4M
		Chalk CaCO_3_				E5/TFF/100:26/CaCO_3_/4M
		Carbon C				E5/TFF/100:26/C/4M
Epidian 53	TFF	Reference samples	7 days	4 months	E53/TFF/4 months	E53/TFF/100:22/4M
		Montmorillonite ZR-2				E53/TFF/100:22/ZR-2/4M
		Chalk CaCO_3_				E53/TFF/100:22/CaCO_3_/4M
		Carbon C				E53/TFF/100:22/C/4M

**Table 5:** Strength test results of the adhesive compositions from the E5/TFF/1 week group.

Adhesive Composition		Maximum Force (N)	Tensile Strength (MPa)	Elongation in Tension (%)
		Mean	Mean	Mean
E5/TFF/100:26/1W	{1}	1923.6 ± 1055.1	13.32 ± 5.82	2.16 ± 0.73
E5/TFF/100:26/ZR-2/1W	{2}	1723.9 ± 525.5	11.67 ± 2.42	1.65 ± 0.41
E5/TFF/100:26/CaCO_3_/1W	{3}	2219.3 ± 608.6	16.48 ± 4.14	2.72 ± 0.64
E5/TFF/100:26/C/1W	{4}	1640.2 ± 635.9	12.07 ± 4.46	2.07 ± 0.79

**Table 6:** Tukey’s honest significant difference test results for the adhesive compositions from the E5/TFF/1 week group.

Adhesive Composition		Tested Group; M—Mean			
		{1} M = 13.32	{2} M = 11.67	{3} M = 16.48	{4} M = 12.07
E5/TFF/100:26/1W	{1}		0.931782	0.671683	0.968197
E5/TFF/100:26/ZR-2/1W	{2}	0.931782		0.338717	0.998947
E5/TFF/100:26/CaCO_3_/1W	{3}	0.671683	0.338717		0.410581
E5/TFF/100:26/C/1W	{4}	0.968197	0.998947	0.410581	

**Table 7:** Strength test results of the adhesive compositions from the E53/TFF/1 week group.

Adhesive Composition		Maximum Force (N)	Tensile Strength (MPa)	Elongation in Tension (%)
		Mean	Mean	Mean
E53/TFF/100:22/1W	{1}	2289.1 ± 303.6	16.84 ± 2.66	3.08 ± 0.34
E53/TFF/100:22/ZR-2/1W	{2}	1432.5 ± 76.7	11.29 ± 1.75	1.95 ± 0.22
E53/TFF/100:22/CaCO_3_/1W	{3}	1987.1 ± 119.5	13.55 ± 0.44	2.31 ± 0.34
E53/TFF/100:22/C/1W	{4}	2149.1 ± 174.5	15.32 ± 2.03	2.29 ± 0.76

**Table 8:** The Kruskal–Wallis rank test results of the strength of the adhesive compositions from the E53/TFF/1 week group.

Adhesive Composition		Tested Group			
		{1} M = 16.84	{2} M = 11.29	{3} M = 13.55	{4} M = 15.32
E53/TFF/100:22/1W	{1}		0.002514	0.222613	1.000000
E53/TFF/100:22/ZR-2/1W	{2}	0.002514		0.893767	0.052888
E53/TFF/100:22/CaCO_3_/1W	{3}	0.222613	0.893767		1.000000
E53/TFF/100:22/C/1W	{4}	1.000000	0.052888	1.000000	

**Table 9:** Strength test results of the adhesive compositions from the E5/TFF/4 month group.

Adhesive Composition		Maximum Force (N)	Tensile Strength (MPa)	Elongation in Tension (%)
		Mean	Mean	Mean
E5/TFF/100:26/4M	{1}	4760.7 ± 1163.7	34.35 ± 9.72	4.59 ± 0.84
E5/TFF/100:26/ZR-2/4M	{2}	3175.9 ± 928.0	22.31 ± 5.71	1.65 ± 0.41
E5/TFF/100:26/CaCO_3_/4M	{3}	1633.0 ± 668.8	13.69 ± 5.93	3.28 ± 0.40
E5/TFF/100:26/C/4M	{4}	1354.3 ± 196.6	10.20 ± 1.83	2.38 ± 0.46

**Table 10:** The Kruskal–Wallis rank test results of the strength of the adhesive compositions from the E5/TFF/4 month group.

Adhesive Composition		Tested Group; M—Mean			
		{1} M = 34.35	{2} M = 22.31	{3} M = 13.69	{4} M = 10.20
E5/TFF/100:26/4M	{1}		1.000000	0.045158	0.005518
E5/TFF/100:26/ZR-2/4M	{2}	1.000000		0.523072	0.112067
E5/TFF/100:26/ CaCO_3_/4M	{3}	0.045158	0.523072		1.000000
E5/TFF/100:26/C/4M	{4}	0.005518	0.112067	1.000000	

**Table 11:** Strength test results of the adhesive compositions from the E53/TFF/4 month group.

Adhesive Composition		Maximum Force (N)	Tensile Strength (MPa)	Elongation in Tension (%)
		Mean	Mean	Mean
E53/TFF/100:22/4M	{1}	3299.7 ± 1671.5	25.19 ± 13.14	3.92 ± 0.56
E53/TFF/100:22/ZR-2/4M	{2}	1986.2 ± 394.8	18.32 ± 3.33	2.49 ± 0.83
E53/TFF/100:22/CaCO_3_/4M	{3}	1555.5 ± 357.7	11.34 ± 2.56	1.67 ± 0.59
E53/TFF/100:22/C/4M	{4}	961.6 ± 281.3	7.83 ± 2.53	0.96 ± 0.50

**Table 12:** The Kruskal–Wallis rank test results of the strength of the adhesive compositions from the E53/TFF/4 month group.

Adhesive Composition		Tested Group; M—Mean			
		{1} M = 25.19	{2} M = 18.32	{3} M = 11.34	{4} M = 7.83
E53/TFF/100:22/4M	{1}		1.000000	0.726683	0.032664
E53/TFF/100:22/C/4M	{2}	1.000000		0.806886	0.038457
E53/TFF/100:22/ZR-2/4M	{3}	0.726683	0.806886		1.000000
E53/TFF/100:22/CaCO_3_/4M	{4}	0.032664	0.038457	1.000000	
